# *Halophytophthora fluviatilis* Pathogenicity and Distribution along a Mediterranean-Subalpine Gradient

**DOI:** 10.3390/jof7020112

**Published:** 2021-02-03

**Authors:** Maria Caballol, Dora Štraus, Héctor Macia, Xavier Ramis, Miguel Á. Redondo, Jonàs Oliva

**Affiliations:** 1 Department of Crop and Forest Sciences, University of Lleida, 25198 Lleida, Spain; dora.straus@udl.cat (D.Š.); hectormacia1993@hotmail.com (H.M.); xavieramis91@gmail.com (X.R.); jonas.oliva@udl.cat (J.O.); 2 Department of Forest Mycology and Plant Pathology, Swedish University of Agricultural Sciences, 750 07 Uppsala, Sweden; miguel.angel.redondo@slu.se; 3 Joint Research Unit CTFC-AGROTECNIO, 25198 Lleida, Spain

**Keywords:** oomycetes, *Quercus suber*, *Alnus glutinosa*, climate change

## Abstract

*Halophytophthora* species have been traditionally regarded as brackish water oomycetes; however, recent reports in inland freshwater call for a better understanding of their ecology and possible pathogenicity. We studied the distribution of *Halophytophthora fluviatilis* in 117 forest streams by metabarcoding river filtrates taken in spring and autumn and by direct isolation from floating leaves. Pathogenicity on six *Fagaceae* species and *Alnus glutinosa* was assessed by stem inoculations. The distribution of *H. fluviatilis* was correlated with high mean annual temperatures (>93.5% of reports in Ta > 12.2 °C) and low precipitation records. *H. fluviatilis* was therefore widely distributed in forest streams in a warm–dry climate, but it was mostly absent in subalpine streams. *H. fluviatilis* was primarily detected in autumn with few findings in spring (28.4% vs. 2.7% of streams). *H. fluviatilis* was able to cause small lesions on some tree species such as *Quercus pubescens*, *Q. suber* and *A. glutinosa*. Our findings suggest that *H. fluviatilis* may be adapted to warm and dry conditions, and that it does not pose a significant threat to the most common Mediterranean broadleaved trees.

## 1. Introduction

Plant pathogens constitute a major threat for natural ecosystems [1]. Amongst them, oomycetes include some of the most devastating genera, like *Phytophthora* and *Pythium*, but they also include less studied genera such as *Halophytophthora*. The genus *Halophytophthora* comprises a group of oomycetes predominantly inhabiting marine ecosystems. They are mostly found in brackish and saline waters where they can tolerate different levels of salinity [2,3,4]. Recent reports challenge the idea that *Halophytophthora* spp. are restricted to saline environments. For instance, *H. vesicula* and *H. spinosa* were isolated in a low-salinity river in Japan [2]. Reeser et al. [5] also recovered *Halophytophthora* species from freshwater streams in Oregon. In Europe, knowledge of freshwater *Halophytophthora* spp. is scarce. Besides several reports in brackish water, only the work by Ďatková [6] recovered *H. fluviatilis* from river streams in Czech Republic. Interestingly, this same species was isolated from freshwater streams in Virginia by Yang et al. [7].

Little is known about the ecology and climatic requirements of freshwater *Halophytophthora* spp. Most of the described *Halophytophthora* species have been isolated in tropical and subtropical regions [8,9,10] or in regions with a warm climate [2,5,11], but recent studies have reported their presence in cold areas of Northern and Central Europe [12,13,14]. In summary, *Halophytophthora* species seem to be more widespread than previously thought, pointing to the need of increasing our understanding of their distribution.

It is currently unknown whether *Halophytophthora* species can act as plant pathogens and cause disease to forest species. Some *Halophytophthora* spp. have been described as early colonizers of fallen mangrove leaves [15], and in other studies they have been shown to play a key role as litter decomposers in mangrove ecosystems [2,3,16]. Nevertheless, a recent study has shown that eelgrass (*Zostera marina*) germination was significantly reduced in seeds infected by *H. zostera* [17], indicating that some species have pathogenic behaviour.

In this research, we studied the distribution of *Halophytophthora fluviatilis* across forest streams along a Mediterranean-subalpine gradient in Catalonia (NE Spain). Distribution was studied by metabarcoding river filtrates taken in spring and autumn using *Phytophthora*-specific primers [18] which amplified many *Phytophthora* spp., in addition to one single cluster of *Halophytophthora*, corresponding to *H. fluviatilis*. Metabarcoding was coupled with an isolation campaign of 28 streams in a N–S gradient from which *H. fluviatilis* isolates were obtained. Pathogenicity of *H. fluviatilis* was assessed by stem inoculations on some abundant *Fagaceae* species in Mediterranean forests in Southern Europe including *Quercus faginea*, *Q. ilex*, *Q. pubescens* and *Q. suber*, and more Central European species, such as *Fagus sylvatica* and *Castanea sativa*. Pathogenicity tests also included *Alnus glutinosa* as a riverbank species with well-known susceptibility to oomycetes.

## 2. Materials and Methods

### 2.1. Streams Survey

**Figure 1:**
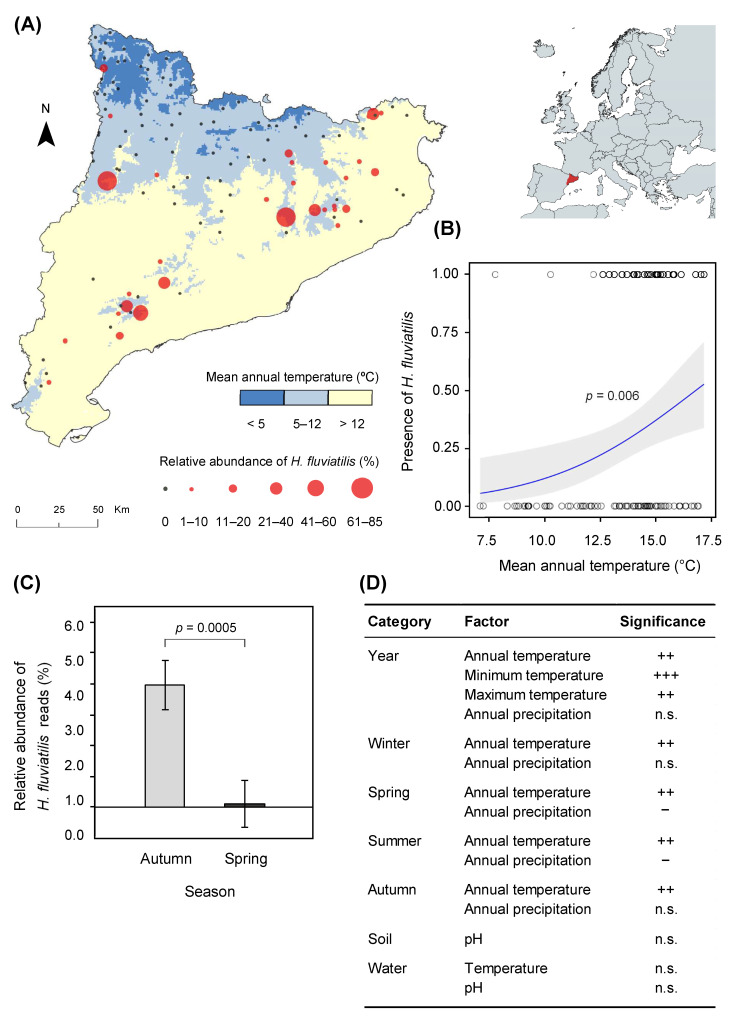
Distribution of *Halophytophthora fluviatilis* in Catalonia (NE Spain) (**A**), and association with mean annual temperature following a logistic regression model for *H. fluviatilis* presence or absence (**B**). Mean relative abundance of reads per sample in autumn and in spring. Error bars represent SE. (**C**). Correlation between presence of *H. fluviatilis* and climatic variables where “+++”and “++” indicate significant positive associations at *p* < 0.001 and *p* < 0.01, respectively (“−“ sign is used for negative associations at *p* < 0.05) (**D**).

We surveyed 117 streams in Catalonia (NE Spain) in two different seasons, autumn 2018 and spring 2019 (Figure 1A). Due to the lack of running water, 5 and 7 streams were missing in autumn and spring, respectively. Our stream network covered a climatic gradient spanning from areas with a subalpine climate with minimum temperatures below 0 °C in winter to coastal areas with mild winters. In the gradient, mean temperatures ranged from 7 to 17.2 °C and precipitation from 386 to 1180 mm. Streams also run through areas with different geology, such as sedimentary limestone or metamorphic bedrock, rendering soils with high and low pH values (from 3.4 to 8.5). Sampling locations were chosen to be easily accessible, containing mainly forest in the upstream catchment area and with minimal presence of agricultural land or urban areas. At each site, 6 L of water was collected with a bucket and filtered through an 8 µm membrane (Merck Millipore, Cork, Ireland) attached to the pump of an agricultural hand sprayer with a polysulfone filter holder as in Redondo et al. [18]. Membranes were replaced every time they became obstructed until all 6 L of water was filtered. Membranes were stored in Petri dishes at 5 °C before transportation to the laboratory and stored at −20 °C until processed for DNA extraction. Pumps were rinsed with 5% sodium hypochlorite and distilled water between samples to avoid cross-contamination. Water temperature and pH were measured at the time of sampling.

### 2.2. Library Preparation for Metabarcoding

Filters were cut in half and DNA was extracted using the NucleoSpin^®^ Soil kit (Macherey-Nagel, Düren, Germany). Libraries were prepared using tagged primers described by Redondo et al. [18], based on the *Phytophthora*-specific primers developed by Drenth et al. [19]. Briefly, three technical replicates of each sample were amplified using the following cycling conditions: an initial denaturation step at 95 °C for 3 min, followed by a number of cycles of 95 °C for 30 s, 60 °C for 30 s, and 72 °C for 1 min, and a final elongation at 72 °C for 6 min. The number of cycles was adjusted for each sample so a faint band indicative of the linear phase of the PCR was observed. PCR products were pooled and cleaned using bead suspension and magnetic separator according to the NucleoMag^®^ NGS Clean-up and Size Select protocol (Macherey-Nagel, Düren, Germany). For library preparation, DNA concentrations were measured with Qubit^TM^, and samples were pooled in equimolar mixtures prior to sequencing. Three pools including a total of 251 samples were sequenced, each one in a SMRT PacBio cell at ScilifeLab (Uppsala, Sweden) yielding a total output of 732,610 reads.

### 2.3. Quality Control and Bioinformatic Analysis of Metabarcoding Data

Sequences were de-multiplexed, filtered and clustered using a similarity threshold of 99.5% with the bioinformatics SCATA pipeline (scata.mykopat.slu.se). Quality filtering was performed by keeping only those reads with mean quality of 2 or higher, containing both primers and tags with a similarity threshold of 90% and being longer than 700 bp. A total of 236,990 reads passed quality control. In order to compare with previous studies in Sweden, the same clustering parameters as in Redondo et al. [18] were used, namely a clustering distance of 0.005 and a 0.90 of minimum length for pairwise alignment. Clustering yielded 405 clusters or operational taxonomic units (OTUs) that were identified by the on-line BLAST search tool in GenBank (ncbi.nlm.nih.gov/genbank/ (accessed on 3 February 2021)). The *H. fluviatilis* cluster was the 10th most abundant OTU (1.9% of all reads). 

### 2.4. Isolation From Leaves

In autumn 2019, we surveyed 28 of those same streams used for metabarcoding distributed in a N–S gradient (Figure S1). Floating and sunken leaves were collected, aiming at collecting as much diversity as possible from each site. Across all sites, the sampled leaves belonged to a wide range of woody and herbaceous plant species, such as *Acer* spp., *Amelanchier* spp., *Buxus* spp., *Celtis* spp., *Cornus* spp., *Corylus* spp., *Crataegus* spp., *Fagus* spp., *Fraxinus* spp., *Hedera* spp., *Lingustrum* spp., *Morus* spp., *Pinus* spp., *Platanus* spp., *Populus* spp., *Prunus* spp., *Quercus* spp., *Rhamnus* spp., *Salix* spp., *Ulmus* spp., *Viburnum* spp. and *Vitis* spp. Some leaves could not be identified. After collection, leaves were placed in Falcon tubes and kept cold in a portable cool box. In the laboratory, leaves were rinsed with distilled water and briefly surface-sterilized with 70% ethanol. Small sections of necrotic leaf tissue were plated onto CMA-PARPBH selective medium [20] and then incubated at 20 °C in darkness for 48–72 h. *Phytophthora*-like hyphae were transferred onto V8A media [21] and stored at 20 °C. A total of 181 isolates were obtained.

### 2.5. Molecular Identification of Isolates

DNA was extracted from isolates via the NaOH fast extraction procedure [22]. The ITS region was amplified using the ITSA2-I2 primers according to PCR conditions described by Samils et al. [23] and sequenced by Macrogen. Isolates were identified as OTUs. Two isolates obtained from two different plots were identified as *H. fluviatilis* (similarity with voucher KF734963 > 99.9%), the remaining 179 isolates were *Phytophthora* species.

### 2.6. Pathogenicity Tests

Pathogenicity trials were run with the *H. fluviatilis* isolates on one-year old seedlings of *Alnus glutinosa* (8.3 mm Ø, stem diameter) and six *Fagaceae* species, namely *Castanea sativa* (7.4 mm Ø), *Fagus sylvatica* (5.5 mm Ø), *Quercus faginea* (5 mm Ø)*, Q. ilex* (5.5 mm Ø), *Q. pubescens* (4.1 mm Ø) and *Q. suber* (4 mm Ø). Inoculations were performed on six replicates per tree species and isolate. Inoculations were carried out by removing the bark and inserting a 5 mm Ø agar plug from a 3-week-old culture of *H. fluviatilis* growing on V8A media. Controls were inoculated with a sterile V8A plug. Wounds were wrapped with Parafilm^®^ and seedlings were kept in a climate chamber for 7 weeks (at 25 °C and 80% relative humidity, and a day/night photoperiod of 16/8 h). After 7 weeks, the vertical length of bark necrosis was measured by gently scraping the bark. The presence of necrosis at the point of inoculation was also noted. Attempts of re-isolating the pathogen were performed by plating pieces of necrotic tissue onto selective media and observing growth after 72 h.

### 2.7. Climatic and Soil Data

Climatic data were obtained from the Meteorological Service of Catalonia (meteo.cat) website and processed with the R package “Meteoland” [24]. The seasonal and yearly average, minimum and maximum temperature and precipitation for the last 44 years were calculated for each sampling plot. Soil pH data were obtained from the plot nearest to the sampled stream site determined from the plot network from Cartographic and Geological Institute of Catalonia website (ICGC; icgc.cat). ICGC plots were usually located no further than 5 km from the sampled stream.

### 2.8. Statistical Analysis

The association between the presence and relative abundance of *H. fluviatilis* and the climatic, water and soil variables was evaluated by logistic regression using the *logit* transformation as a link function and assuming a binomial distribution. Overdispersion of relative abundance data was accommodated by using a *quasibinomial* distribution. Logistic regression analyses were carried out with the glm function of the R package “Tidyverse” [25]. The percentage of *H. fluviatilis* reads was compared between autumn and spring by a mixed model using location as a random factor. Lesion length obtained in the inoculation test was compared between inoculated and non-inoculated seedlings across tree species with an ANOVA in JMP Pro (version 15.2.0; SAS Institute Inc., Cary, NC, USA). For each seedling, we averaged the lesion above and below the inoculation point. In order to meet the normality assumption, lesion length was log-transformed. 

## 3. Results

### 3.1. Halophytophthora fluviatilis Distribution

*Halophytophthora fluviatilis* was found widespread across the sampled area (Figure 1A). *H. fluviatilis* was more frequently detected in autumn than in spring (28.4% vs. 2.7% of the surveyed streams, respectively) (Figure 1C). It was restricted to streams in warm and dry regions. In 93.5% of the cases in which it was found, the average annual temperature of the area was higher than 12.2 °C (Figure 1B). The yearly mean temperature as well as the mean temperature of winter, spring, summer and autumn were found to be positively correlated with the presence of *H. fluviatilis*. Precipitation in spring and summer months was negatively associated with the presence of *H. fluviatilis*. Neither the temperature and pH of stream water at the time of sampling nor the soil pH were significant for the presence of *H. fluviatilis* (Figure 1D). 

Out of the surveyed streams in the N–S gradient, *H. fluviatilis* was detected by metabarcoding in about a third of them (28.6%). However, isolates could only be obtained from two streams in the gradient from which *H. fluviatilis* had not been detected by metabarcoding (Figure S1). One of the isolates was obtained from a leaf of *Platanus* spp., while the other from a leaf that was too decomposed to be identified.

### 3.2. Pathogenicity Assessment

**Figure 2:**
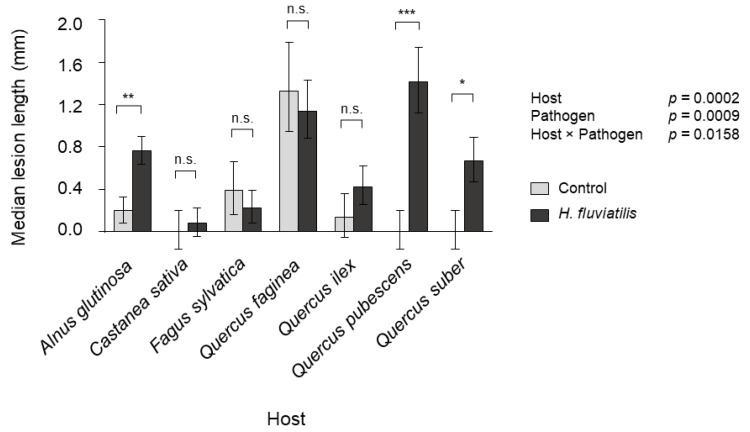
Pathogenicity of *H. fluviatilis* in *Alnus glutinosa* and *Fagaceae* species measured as lesion length 7 weeks after stem inoculations. Asterisks depict significant differences (* *p* < 0.05, ** *p* < 0.01, *** *p* < 0.0001). “n.s.” indicates non-significant differences at *p* < 0.05. Error bars represent SE.

*Halophytophthora fluviatilis* was weakly pathogenic towards some of the studied species (Figure 2). Two months after inoculation, *Alnus glutinosa*, *Quercus pubescens* and *Q. suber* showed lesions that were significantly larger than controls (Figure 2). 

## 4. Discussion

The genus *Halophytophthora* includes a group of oomycetes predominantly inhabiting marine ecosystems. However, recent reports in inland freshwater locations show that some *Halophytophthora* species seem to be adapted to these ecosystems and indicate a need to understand their ecology and possible pathogenicity for common forest trees. We studied the distribution of *H. fluviatilis* in a network of 117 streams across a climatic gradient in Catalonia (NE Spain) and assessed its pathogenicity on common broadleaved tree species. Our findings suggest that climate may be limiting *H. fluviatilis* presence; however, the biological mechanisms underlying its susceptibility to cold and rainy conditions require further study. At first glance, *H. fluviatilis* does not seem to pose a major threat to main forest species in this area. However, further understanding the host range and the pathogenic status of *H. fluviatilis* may be useful to clarify the role of this oomycete in Mediterranean-subalpine ecosystems. 

The presence of *H. fluviatilis* in our network of streams was restricted to warm and dry areas. Climatic factors have been shown to limit the establishment and distribution of some oomycetes [26]. In our study, *H. fluviatilis* was mainly detected in streams of regions with a temperature higher than ca. 12 °C, and its presence was negatively associated with precipitation in spring and summer months. In the studied region, precipitation is negatively correlated with temperature (r = −0.77); therefore, it is difficult to disentangle one effect from the other. However, when restricting our analysis to areas with a warm climate (data not shown), there was no association between precipitation and presence or relative abundance of *H. fluviatilis*, thereby indicating a temperature effect. Given the aquatic lifestyle of *H. fluviatilis*, our results agree with Redondo et al. [18] who showed that temperature, and not precipitation, seems to be the main environmental filter for *Phytophthora* species with an aquatic lifestyle in contrast with those found in terrestrial ecosystems. Indeed, one would expect *H. fluviatilis* to be resistant to cold because of its functional traits [27], such as homothallic nature and the capacity to produce resistant survival structures such as hyphal swellings or oospores [7]. Nevertheless, the high optimum temperature of this species (25 °C) already indicated preference for warm environments [18,28], and its ability to produce resistant structures may be an adaptation to drought [29,30]. A study conducted in Swedish rivers using the same metabarcoding approach as ours did not detect *Halophytophthora* spp. [18], indicating that cold temperatures may pose a constraining factor for this species. Streams in warm and dry areas usually dry up in summer, and, therefore, it might be that *H. fluviatilis* resistance traits could be adaptations to conditions experienced in ephemeral waterbodies, i.e., periodic drying where resting structures will enable survival until the next rain event. Ephemeral waterbodies also experience fluctuations in salinity that might provide a niche for *H. fluviatilis* which is adapted to slight saline conditions [7].

The observed distribution of *H. fluviatilis* could also be explained by competitive exclusion, i.e., *H. fluviatilis* is outcompeted in colder environments by other organisms. It is not possible to discard this possibility without a deeper knowledge of the niche of *H. fluviatilis* and the associated microbial community. In our study, a single *Halophytophthora* OTU was present in the metabarcoding data, which at least discards possible competition within the same genus, as seen for other *Phytophthora* species [26]. However, competition with other oomycetes would be possible and could be further studied. The same occurs concerning the lack of information about its putative hosts and whether this could be indirectly limiting its distribution.

In our study, metabarcoding enabled a more detailed analysis of the *H. fluviatilis* community dynamics than isolations from floating and sunken leaves. While *H. fluviatilis* was detected by metabarcoding in ca. a third of the streams in a N–S gradient, isolation was only successful in 7.1% of the rivers after screening almost two hundred isolates. Metabarcoding seems to be a good technique for studying the distribution of *H. fluviatilis* and could be applied to other environments to further increase our understanding of its ecology. 

We also tested whether *H. fluviatilis* could be pathogenic to forest species. While our study showed that *H. fluviatilis* may be weakly pathogenic to *Q. pubescens*, *Q. suber* and *A. glutinosa*, these results should be taken with caution. Even if necrosis at the inoculation point occurred and lesions were significantly larger than the control, it is unclear whether *H. fluviatilis* could cause disease in field conditions. In parallel with the *H. fluviatilis* pathogenicity assessment, several known pathogenic *Phytophthora* species such as *P. cambivora* caused much larger lesions than *H. fluviatilis* in a study with the same plant material and tree species (7.9 mm vs. 0.6 mm).

## 5. Conclusions

In this study, we showed that climatic factors seem to restrict the distribution of *H. fluviatilis* in a Mediterranean-subalpine context. *H. fluviatilis* was restricted to stream sites with an average temperature higher than 12.2 °C and was rare in streams flowing from subalpine areas with heavy rainfall and low mean annual temperatures. We also found that *H. fluviatilis* could be slightly pathogenic to *Q. pubescens*, *Q. suber* and *A. glutinosa*. These results provide a better understanding of *H. fluviatilis* ecology, but more research will be needed to understand its ecology and functioning in freshwater ecosystems.

## Data Availability

The data of *H. fluviatilis* pathogenicity and distribution are deposited in Figshare: doi.org/10.6084/m9.figshare.13691191.v1 [31].

## Supplementary Materials

The following are available online at https://www.mdpi.com/2309-608X/7/2/112/s1, Figure S1: Location of sampling sites used for isolation.
