# Supplementary material for: Halophytophthora fluviatilis Pathogenicity and Distribution along a Mediterranean-Subalpine Gradient
Source: J Fungi (Basel). 2021 Feb 3;7(2):112. doi: 10.3390/jof7020112 (PMC7913473; doi:10.3390/jof7020112)
Supplement: Supplementary file 1 [file jof-07-00112-s001.zip › Figure S1.pptx]

## Slide 1
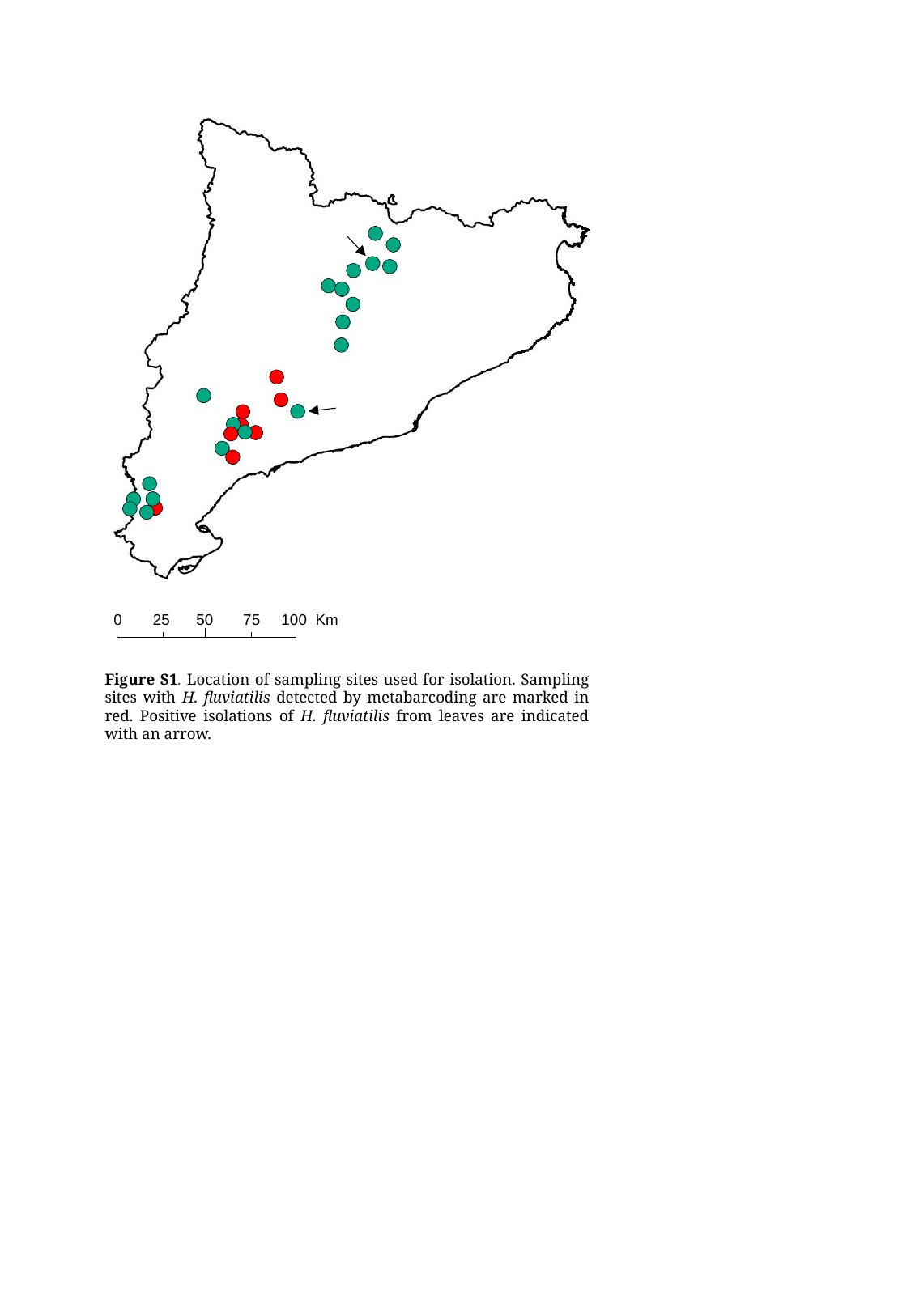

0 25 50 75 100 Km
Figure S1. Location of sampling sites used for isolation. Sampling sites with H. fluviatilis detected by metabarcoding are marked in red. Positive isolations of H. fluviatilis from leaves are indicated with an arrow.
